# Research-based PAM50 signature and long-term breast cancer survival

**DOI:** 10.1007/s10549-019-05446-y

**Published:** 2019-09-21

**Authors:** Minya Pu, Karen Messer, Sherri R. Davies, Tammi L. Vickery, Emily Pittman, Barbara A. Parker, Matthew J. Ellis, Shirley W. Flatt, Catherine R. Marinac, Sandahl H. Nelson, Elaine R. Mardis, John P. Pierce, Loki Natarajan

**Affiliations:** 1 Moores Cancer Center, University of California, San Diego, San Diego, CA USA; 2 Department of Family Medicine and Public Health, University of California, San Diego, 3855 Health Sciences Drive #0901, La Jolla, CA 92093-0901 USA; 3 Department of Medicine, Washington University St. Louis, St. Louis, MO USA; 4 Washington University St. Louis, McDonnell Genome Institute, St. Louis, MO USA; 5 Department of Medicine, University of California, San Diego, San Diego, CA USA; 6 Baylor College of Medicine, Lester and Sue Smith Breast Center, Houston, TX USA; 7 Nationwide Children’s Hospital, Institute for Genomic Medicine, Columbus, OH USA; 8 Division of Population Sciences, Department of Medical Oncology, Dana-Farber Cancer Institute, Boston, MA USA; 9 Department of Epidemiology, Harvard T.H. Chan School of Public Health, Boston, MA USA; 10 Precision for Medicine, San Diego, CA USA

**Keywords:** Breast cancer, Long-term survival, Gene signatures, Hypoxia, PAM50 subtypes, Prognostic modeling

## Abstract

**Purpose:**

Multi-gene signatures provide biological insight and risk stratification in breast cancer. Intrinsic molecular subtypes defined by mRNA expression of 50 genes (PAM50) are prognostic in hormone-receptor positive postmenopausal breast cancer. Yet, for 25–40% in the PAM50 intermediate risk group, long-term risk remains uncertain. Our study aimed to (i) test the long-term prognostic value of the PAM50 signature in pre- and post-menopausal breast cancer; (ii) investigate if the PAM50 model could be improved by addition of other mRNAs implicated in oncogenesis.

**Methods:**

We used archived FFPE samples from 1723 breast cancer survivors; high quality reads were obtained on 1253 samples. Transcript expression was quantified using a custom codeset with probes for > 100 targets. Cox models assessed gene signatures for breast cancer relapse and survival.

**Results:**

Over 15 + years of follow-up, PAM50 subtypes were (*P* < 0.01) associated with breast cancer outcomes after accounting for tumor stage, grade and age at diagnosis. Results did not differ by menopausal status at diagnosis. Women with Luminal B (versus Luminal A) subtype had a > 60% higher hazard. Addition of a 13-gene hypoxia signature improved prognostication with > 40% higher hazard in the highest vs lowest hypoxia tertiles.

**Conclusions:**

PAM50 intrinsic subtypes were independently prognostic for long-term breast cancer survival, irrespective of menopausal status. Addition of hypoxia signatures improved risk prediction. If replicated, incorporating the 13-gene hypoxia signature into the existing PAM50 risk assessment tool, may refine risk stratification and further clarify treatment for breast cancer.

**Electronic supplementary material:**

The online version of this article (10.1007/s10549-019-05446-y) contains supplementary material, which is available to authorized users.

## Introduction

Breast cancer is a heterogeneous disease with large variations in relapse rates even among patients with similar clinical profiles. Several multi-gene prognostic tests are included in national and international guidelines to assist in determining risk of relapse and to better inform treatment decisions [1–7]. Even so, more information is needed regarding how these tests perform in well-characterized cohorts of breast cancer patients with long-term follow-up [8].

The breast cancer intrinsic molecular subtypes, defined by mRNA expression of 50 genes (PAM50), have been shown to improve prognostication significantly compared to standard tumor characteristics and other genomic signatures [3, 9–15]. The related proprietary Prosigna gene signature, FDA-approved in 2013, was validated to estimate relapse risk in postmenopausal women with early stage, hormone-receptor positive breast tumors, but has not been validated in pre-menopausal women [16]. In addition, for the research-based PAM50 signature and the Prosigna test [16, 17], an estimated 25–40% of patients are characterized as having “intermediate risk,” and for these patients, the long-term risk of relapse remains uncertain.

Gene expression studies have identified hundreds of mRNAs implicated in breast cancer. One approach to improving predictive accuracy of existing signatures, such as PAM50, is to evaluate the added prognostic value of independent biomarkers. However, given the large number of potential candidate biomarkers, false positives pose a serious obstacle. Consideration of a priori genes with known oncogenic function could partially mitigate these problems. Hypoxia impacts tumor progression, and hypoxia-related genes are prognostic in breast cancer [18, 19]; hence addition of hypoxia genes to a PAM50 model could elucidate their added prognostic value. Another approach to reduce false discoveries is to use modern statistical methods, such as penalized regression [20], to select prognostic markers from a large candidate list. These methods reduce overfitting of prognostic models and improve future model performance, especially for models with a large number of candidate markers.

Women with ER+ breast cancer continue to relapse 15 years after their primary diagnosis [21, 22]. Treatment options and survival for women with Her2+ tumors have vastly improved since the approval of trastuzumab [23]. However, the Her2+ subgroup comprises < 25% of breast cancers, while women with ER+/Her2− tumors constitute a majority of all breast cancers. Recent research has focused on this ER+/Her2− group to evaluate biomarker-driven treatment approaches in this subgroup [14, 24–27]. Thus, evaluating long-term prognostic value of PAM50 subtypes in the ER+/Her2− subgroup could further clarify its clinical utility. Similarly, although the PAM50-Prosigna signature was originally validated in postmenopausal breast cancer survivors, it may also be prognostic in pre-menopausal breast cancer. Thus it is important to test if the prognostic value of this signature varies by menopausal status at diagnosis.

In this study, using > 1200 archived tumor samples from a large breast cancer cohort with 15 + years of follow-up [28], we investigated genomic predictors of long-term disease-free survival and breast cancer mortality. We previously examined an a priori set of microRNA targets in this sample [29]. The focus in the current work was to examine mRNA expression and breast cancer outcomes, and as a first step we investigated the original research-use PAM50 signature which classified tumors into five distinct subtypes: Luminal A, Luminal B, Basal, Her2-enriched, and normal-like [10]. We also tested a refinement [30], which adds a sixth subtype, the *claudin*-*low* cluster. This claudin subtype is characterized by low luminal, Her2, proliferation expression, high immune response, and epithelial-to-mesenchymal transition expression. Second, we tested if addition of other mRNAs improved prediction via (i) a *targeted* approach with two a priori hypoxia signatures [18, 19] (ii) an *unbiased* approach with penalized regression used to select the most prognostic features from among clinical factors, PAM50 subtypes, and 61 individual mRNAs. Third, we investigated PAM50-prognostic value in the ER+/Her2− subgroup, and if subtype-outcome associations differed by menopausal status at diagnosis.

## Methods

### Study sample

The Women’s Healthy Eating and Living (WHEL) Study, a randomized controlled trial of 3088 breast cancer survivors, tested whether a high fruit/vegetable diet reduced recurrence rates in early stage breast cancer [28, 31]. Women within 4 years of diagnosis with primary operable invasive Stage I (≥ 1 cm), Stage II or Stage III breast carcinoma [32], aged 18 to 70 years at diagnosis, and completed primary treatment for breast cancer were recruited between 1995 and 2000. We obtained IRB approval from participating institutions, and written informed consent from all participants, including for genomic analyses. Formalin-fixed paraffin-embedded (FFPE) tissue samples from the primary tumor were available for 60% (*n* = 1723) of the WHEL cohort. The final analysis for this investigation was based on 1253 participants. As the dietary intervention produced no group effect [28], we treated the study population as a single cohort.

### Study endpoints

In this study, we evaluated two outcomes (i) a breast cancer event (locoregional recurrence, metastasis, or contralateral), and (ii) death from breast cancer. Events were independently adjudicated by two breast oncologists. Carcinoma in situ was not counted as a breast cancer event. The WHEL study ceased active surveillance for cancer events in 2010. Since then, deaths were ascertained by annual searches of the National Death Index. Time from diagnosis to a second breast cancer event defined the disease-free survival outcome; time from diagnosis to breast cancer death defined the breast cancer survival outcome. Time-to-event was censored at death (from non-breast cancer causes), last contact or end of follow-up (2010 for breast cancer events, 2015 for death).

### Nucleic acid extraction

Details on our assay were previously published [29]; for the sake of completeness, we briefly summarize the approach here. Archival tumor blocks were prepared into histological sections. (5 µm each). One slide was stained with hematoxylin and eosin for histopathological review and to guide tumor macrodissection from four unstained sections. The remaining unstained slides from samples with ≥ 40% tumor cellularity were incubated at 65 °C for 30 min and deparaffinized using Citrisolv (Fisher Scientific, Pittsburgh, PA) followed by ethanol wash. Tumor tissues were macrodissected into RNAse-free microfuge tubes, and nucleic acids isolated using the Qiagen AllPrep FFPE kit (#80234). Manufacturer’s instructions were followed with the exception that the proteinase K digestion step was extended to an overnight incubation for DNA isolation. Total RNA and DNA were quantified using the Invitrogen Qubit and corresponding quantification kits. DNA pellets were stored at − 80° C for future use.

### mRNA quantification

Transcript expression was quantified with 250 ng total RNA using the NanoString nCounter analysis system with a custom miRGE CodeSet containing probes for 123 gene expression targets (see Table S1). This gene set was chosen primarily to include targets in the PAM50, claudin-low, VEGF13 and VEGF15 signatures. Assay reactions were assembled per manufacturer’s specifications (NanoString Technologies, INC Seattle, WA).

### Gene signatures

*PAM50:* Expression of the PAM50 (Table S1) genes were normalized to negative and positive controls, and standardized to five housekeepers, as per standard practice [10]. The published PAM50 algorithm [10] was used to classify each subject into an intrinsic subtype: Luminal A, Luminal B, basal-like, Her2-enriched, normal-like. Prior to implementing this algorithm, mRNA values were platform-adjusted [33]. Risk-of-recurrence scores incorporating tumor size and proliferation index (ROR-PT) were calculated, and categorized into low, medium and high risk strata [10].

#### Claudin-low

A 30-gene set was used to classify tumors to the claudin-low versus non-claudin subtypes. Centroids were derived using publicly available microarray data [30] previously used to train the claudin-low signature. Spearman correlations between these centroids and the 30-gene expression values for each tumor were calculated. Tumors with correlation ≥ 0.4 with the claudin-low centroid were classified as claudin-low; else they were classified as non-claudin-low type. Non-claudin-low tumors were assigned their PAM50 class as per the research-based subtype call [10]. Again, Zhao’s method [33] was used for platform adjustment.

#### Hypoxia signatures

We evaluated two hypoxia signatures. A 13-gene VEGF signature [19], VEGF13, and a 15-gene network-based hypoxia metagene [18], VEGF15 (Table S1). After standardizing to housekeepers, VEGF13 and VEGF15 were calculated as the average of log-transformed 13- and 15- gene expression values, respectively. Tertiles of these scores were used to create low, medium or high risk groups. The VEGF13 and VEG15 signatures have only three mRNAs in common, thus potentially provide independent prognostic information.

#### Other genes

We also obtained mRNA expression for six genes (Table S1) implicated in tumor invasion, proliferation, or other oncogenic function.

### Statistical approach

#### Prognostic modeling

Associations between PAM50 subtypes and tumor characteristics were investigated via ANOVA and Chi square tests. Prognostic value of PAM50 subtypes and ROR-PT risk categories for breast cancer outcomes were assessed via Kaplan–Meier plots, and unadjusted and adjusted Cox models, adjusted for clinical variables, namely age at diagnosis, tumor stage, and grade. The VEGF13 or VEGF15 signatures were then added to the model which included clinical variables (age at diagnosis, stage, grade) and PAM50 subtypes. We used delayed entry models [34] to account for varying times from cancer diagnosis to study entry. Likelihood ratio tests and Akaike information criteria (AIC) were used to compare models.

#### Variable selection

We used penalized regression for unbiased variable selection. We included all variables, namely, clinical factors, PAM50 subtypes, and 61 individual mRNAs (including 25 hypoxia, 30 claudin-low genes) in the model and used penalized Cox regression implemented via a *lasso penalty* [20]. The tuning parameter λ, which controls overfitting, was chosen by 10-fold cross-validation to minimize model deviance.

The statistical software package R [35] was used for all statistical analysis.

## Results

### PAM50 and clinical and demographic characteristics

Of the 1723 FFPE samples, 25% had low tumor cellularity or low RNA content and could not be assayed. Gene expression was obtained on 1291 samples; of these 38 were eliminated due to outliers or poor-quality reads. The final WHEL-PAM50 sample comprised of *N* = 1253 breast cancer survivors. Study characteristics were similar to the parent WHEL Study (*N* = 3088) [28]. Women were at an average of 50 years at cancer diagnosis: 85% were White, 36% had Stage I, and 46% had Stage II tumors, three-quarters had ER+ histopathology, and 16% had triple negative histopathology (Table 1). In addition, 78.5% of them were post-menopausal. There were 303 breast cancer events (locoregional recurrence, metastasis, or contralateral breast cancer) and 219 deaths due to breast cancer.

**Table 1:** Participant demographic and clinical characteristics at study entry (*N* = 1253)

Age at breast cancer diagnosis	
Median (range)	50 (27–70)
Race/Ethnicity *N* (%)	
White	1060 (84.6%)
Black	45 (3.6%)
Hispanic	85 (6.8%)
Asian	31 (2.5%)
Other	32 (2.6%)
Stage *N* (%)	
I	453 (36.2%)
IIA	432 (34.5%)
IIB	144 (11.5%)
IIIA	166 (13.2%)
IIIC	58 (4.6%)
Nodal status *N* (%)	
Negative	702 (56%)
Positive	551 (44%)
Tumor size (cm)	
Mean (SD)	2.3 (1.44)
Grade *N* (%)	
Poorly differentiated	497 (39.7%)
Moderately differentiated	496 (39.6%)
Well differentiated	159 (12.7%)
Unspecified	101 (8.1%)
Histopathology *N* (%)	
ER+	909 (73.7%)
PR+	809 (66.4%)
Her2+	217 (17.3%)
Triple negative	199 (15.9%)
Years diagnosis to study entry	
Median (25th, 75th %-iles)	1.8 (1.03, 2.8)
Chemotherapy and Anti-estrogen therapy *N* (%)	
Yes, yes	590 (47.1%)
Yes, no	314 (25.1%)
No, yes	258 (20.6%)
No, no	76 (6.1%)
Yes, unknown	5 (0.4%)
No, unknown	9 (0.7%)
Outcomes	
Breast cancer events (*N*)	303
Disease-free survival (years)	
Median (25th, 75th)%-iles	9.5 (6.7, 11.3)
Breast cancer deaths (*N*)	219
Breast cancer survival (years)	
Median (25th, 75th)%-iles	16.8 (15.3, 18.2)

PAM50 subtype distributions were 45% Luminal A, 23% Luminal B, 18% basal, 11% Her2-enriched, and 3% normal-like. Subtypes were significantly associated with clinical characteristics and menopausal status at diagnosis (Table 2). The proportion of Luminal A tumors decreased with increasing tumor stage (56% Stage 1, 29% Stage IIIC). Also, 25% Stage IIIC vs 16% Stage I tumors were basal. Poorly differentiated tumors had a high proportion of basal subtype. Luminal A subtype tumors were more prevalent, while basal and Luminal B subtypes were less prevalent in women who were postmenopausal at diagnosis compared to women who were pre-menopausal at diagnosis (Table 2). As expected (Table S2), basal subtype constituted 77% of triple negative tumors, while ER+ tumors were predominantly luminal (55% Luminal A, 29% Luminal B). The subtype distribution for ER+/Her2− tumors was similar to ER+ tumors, whereas ER+/Her2+ tumors were split across Her2-enriched (34%), Luminal A (29%) and B subtypes (31%). Due to low prevalence, the “normal-like” subtype was excluded from the outcome analysis.

**Table 2:** Distribution of PAM50 subtypes by clinical characteristics

		Luminal A %	Luminal B %	Basal-like %	Her2%	Normal %	*P*
	*N*	564	284	225	139	41	
Cancer stage							< 0.0001
I	453	55.6	17.7	15.9	8.2	2.6	
IIA	432	42.4	22.9	20.1	11.1	3.5	
IIB	144	34.7	26.4	20.1	15.3	3.5	
IIIA	166	37.4	31.3	13.3	13.9	4.2	
IIIC	58	29.3	25.9	25.9	15.5	3.5	
Tumor grade							< 0.0001
Well-differentiated	159	80.5	12	2.5	1.3	3.8	
Moderately-diff	496	57.3	26.6	4.8	7.9	3.4	
Poorly diff	497	17.9	23.9	37.4	18.1	2.6	
Unspecified	101	62.4	13.9	10.9	7.9	5	
Mean age at diagnosis (SE)		52.8 (0.4)	50.8 (0.5)	48.2 (0.6)	50.5 (0.8)	49.6 (1.2)	<0.0001
Menopausal status at diagnosis							0.02
Pre-menopausal		40.3	25	19.9	10.9	3.9	
Post-menopausal		49.7	20.3	16.3	11.3	2.7	

### PAM50 and breast cancer outcomes

**Fig. 1:**
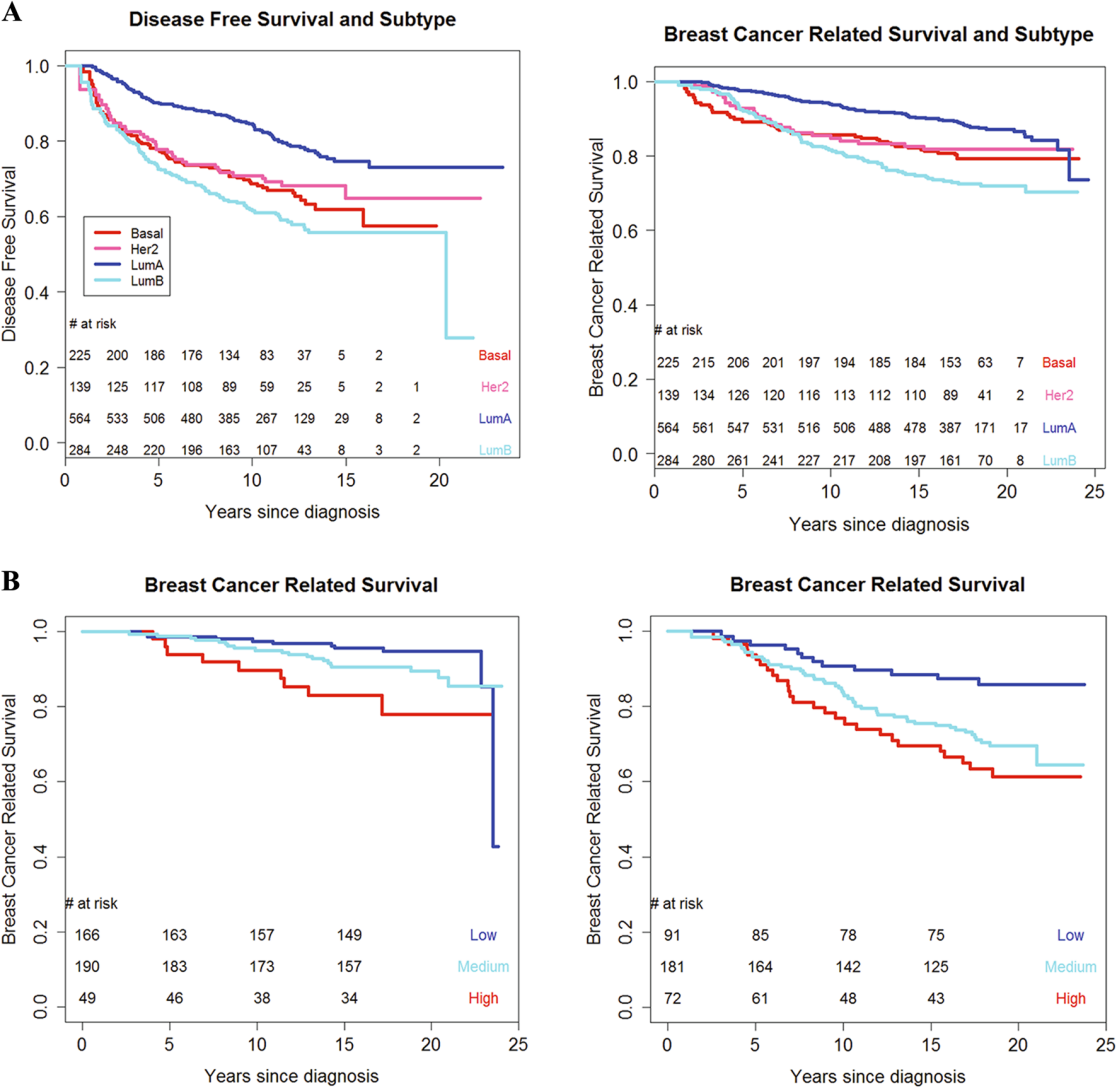
**a** Kaplan–Meier curve of PAM50 subtype and Disease-free survival (left, *P* < 0.001) and Breast cancer survival (right *P* < 0.001). **b** Kaplan–Meier curve of ROR-PT category and Breast cancer survival by nodal status (left node-negative *P* = 0.007; right node-positive *P* = 0.003). *P*-value based on likelihood ratio test comparing null (unadjusted) to PAM50 model

Kaplan–Meier curves (Fig. 1) for the four subtypes were well separated (*P* < 0.001 for disease-free and breast cancer survival). Luminal A subtype had the best outcomes with 10-year rate of 0.85 (95% CI 0.81–0.88). Interestingly, the 10-year rate was the lowest in the Luminal B group—0.61 (95% CI 0.55–0.69), and basal and Her2-enriched tumors had intermediate rates of 0.69 (95% CI 0.62–0.76) and 0.71 (95% CI 0.60–0.84), respectively.

Multivariable adjusted Cox models (Table 3) showed similar effects, with a 60% higher hazard of a breast cancer event or death for the Luminal B versus Luminal A subtype. Likelihood ratio tests confirmed the prognostic value of the PAM50 signature over clinical factors (*P* < 0.01).

**Table 3:** Associations between PAM50 subtypes and hypoxia signatures with breast cancer outcomes: Multiple regression survival analysis*

	Disease-free survival (*N* = 295 relapse events)*	Breast cancer survival (*N* = 212 breast cancer deaths)*
PAM50 subtype^a^	HR (95% CI)	HR (95% CI)
Luminal A (ref)	1.0	1.0
Basal	1.24 (0.87, 1.78)	1.01 (0.65, 1.55)
Her2	0.98 (0.65, 1.49)	0.91 (0.56, 1.49)
Luminal B	1.60 (1.19, 2.13)	1.68 (1.20, 2.35)
Model comparison: clinical vs (PAM50 + clinical)		
Likelihood ratio test: Chi square statistic	11.7	12.9
Degrees of freedom	3	3
*P*-value	0.009	0.005
VEGF13 signature^b^		
Low (ref)	1.00	1.00
Medium	1.33 (0.99, 1.78)	1.27 (0.90, 1.79)
High	1.48 (1.08, 2.02)	1.41 (0.98, 2.03)
Model comparison: (PAM50 + clinical) vs (PAM50 + clinical +VEGF13)		
Likelihood ratio test: chi square statistic	6.2	3.6
Degrees of freedom	2	2
*P*-value	0.04	0.16
VEGF15 signature^b^		
Low (ref)	1.00	1.00
Medium	0.92 (0.68, 1.24)	0.92 (0.65, 1.31)
High	1.33 (0.99, 1.78)	1.33(0.94,1.87)
Model comparison: (PAM50 + clinical) vs (PAM50 + clinical +VEGF15)		
Likelihood ratio test: chi square statistic	6.7	4.8
Degrees of freedom	2	2
*P*-value	0.03	0.09

*Subjects who were classified as normal-like subtype were excluded from this analysis

^a^Model adjusted for age at diagnosis, tumor grade, tumor stage

^b^Model adjusted for age at diagnosis, tumor grade, tumor stage, PAM50 subtype

### Hypoxia gene signatures and prognosis

Adding VEGF13 to the PAM50 model improved prognostication (likelihood ratio test *P* = 0.04), with > 40% higher hazard of breast cancer events for the highest vs lowest tertile (Table 3). Hazard ratios were similar for VEGF13 and breast cancer deaths, although results were not statistically significant (Table 3). The results for VEGF15 were similar to VEGF13 (Table 3).

### Identifying individual prognostic mRNAs

The penalized regression [20] analysis for disease-free survival identified tumor stage and PAM50 subtype as the most prognostic variables. Additional selected features were FLVCR2, which encodes a calcium transporter protein, and FABP5, implicated in fatty acid binding, both in the VEGF13 signature, justifying our a priori models. These four variables were also selected in the breast cancer mortality model, in addition to ANGPTL4, a VEGF-13 marker implicated in angiogenesis, and SPINT1, a claudin-low feature involved in epithelial cell differentiation. The estimated hazard ratios with 95% CIs of the selected features are presented Table 4; the 95% CIs do not account for the selection process, and should be interpreted with caution.

**Table 4:** Transcripts associated with breast cancer outcomes: results of penalized regression

Selected mRNAs	Disease-free survival^a^ Hazard ratio^b^ (95% CI)	Breast cancer mortality^a^ Hazard ratio^b^ (95% CI)
FLVCR2	0.85 (0.77, 0.93)	0.8 (0.70, 0.90)
FABP5	1.14 (1.06, 1.23)	1.13 (1.04, 1.24)
ANGPTL4	Not selected	1.09 (1.02, 1.17)
SPINT1	Not selected	1.11 (1.01, 1.22)

^a^Models also adjusted for tumor stage, grade, and PAM50 subtype

^b^Hazard ratio represents increase in hazard per unit increase in (log2)-mRNA

### Subgroups and refinements

Results did not differ by menopausal status at diagnosis (subtype*menopausal interaction *P* value ≥ 0.3). Among pre-menopausal women, adjusted hazard ratios for disease-free survival were: 1.24 for basal, 0.96 for Her2-enriched, and 1.55 for Luminal B subtypes compared to Luminal A subtypes, and for postmenopausal women the corresponding hazard ratios were 1.25 for basal, 1.05 for Her2-enriched, and 1.63 for Luminal B subtypes. Hazard ratios for PAM50 subtypes also did not differ by age categories: < 50 versus ≥ 50 years at diagnosis.

With the inclusion of 30 claudin genes, 5% (*N* = 64) were classified as claudin-low. Of these, 59% were previously classified as basal, 22% as luminal A, and 11% as normal-subtype. The 10-year disease-free survival rate (95% CI) for the claudin-low group (Fig S1) was 0.81 (0.71, 0.94); 10-year rates for the other subtypes, after incorporation of the claudin-low subtype, were similar to the original PAM50 calls. Inclusion of the claudin-low subtype did not improve model fit: Akaike information criterion statistic was 3839.5 for the claudin-low-added versus 3837.9 for the standard PAM50 signatures.

Further investigation of PAM50 risk scores in the ER+/Her2− subgroup confirmed that ROR-PT risk categories were associated with disease-free survival and breast cancer death (Fig. 1b). We stratified plots by nodal status given its established prognostic value and key role in determining course of breast cancer treatment [8]. Ten-year breast cancer mortality rates were 3%, 5%, and 10% for the low, medium, high ROR-PT categories among node-negative (*P* = 0.007), and 10%, 16% and 23% in the low, medium, high ROR-PT groups among node-positive (*P* = 0.003) survivors. Similarly, for disease-free survival in the node-negative stratum, 10-year event rates were 10%, 13%, and 32% (*P* = 0.05) for the low, medium, and high risk groups, respectively. For node-positive patients, the corresponding 10-year event rates were 19%, 36%, and 44% (*P* = 0.02). These results are concordant with previous findings on risk separation by ROR-PT categories and breast cancer outcomes in ER+/Her2− breast cancer [36].

## Discussion

In this study, we confirmed the prognostic value of PAM50 subtypes over clinical factors in an independent breast cancer cohort with long-term follow-up. Our results did not differ by age or menopausal status at diagnosis, suggesting that PAM50 subtypes are prognostic across the age spectrum. In recent years, a plethora of gene markers implicated in breast cancer have been identified. Hypoxia impacts tumor progression, and hence we investigated two hypoxia-related gene signatures [18, 19]. These signatures added significant prognostic value to the model with clinical variables (age at diagnosis, stage, grade) and PAM50 subtypes: participants with high levels of the hypoxia signature (i.e., highest tertile) had 30–40% increased hazard for relapse compared to those with lower levels (bottom tertile). Replication of this finding in independent cohorts and additional research on incorporating these signatures for clinical use is needed.

We evaluated the claudin-low subtype [30] and found that incorporation of this refinement did not improve prognostication for disease-free or breast cancer survival in our sample. Our results are similar to Dias [37], but differ from other studies [30], which found worse survival in the claudin-low group. There are clinical and treatment differences between studies, which could explain these discrepancies. Only 5% (*N* = 64) of tumors in our study were classified as claudin-low, limiting our ability to conduct further sensitivity analysis on this subgroup.

An important finding is the consistently worse survival rates in the Luminal B subtype irrespective of menopausal status. Women with this subtype, which constitutes ~ 25% of breast cancers, continued to experience poor outcomes even 15 years after diagnosis. Identifying genomic markers, treatments and modifiable risk factors specific to this subgroup could improve long-term outcomes for a large proportion of breast cancer survivors.

Our study has many strengths. The study sample comprised a large well-characterized clinical cohort with over 15 years follow-up including both pre-and post-menopausal women of all hormonal and Her2 subtypes. We obtained high-quality assays using the validated Nanostring platform, and derived subtype calls of > 95% confidence for 90% of our sample. We used rigorous statistical approaches for model development and implemented modern penalized regression methods for unbiased variable selection. There are limitations. Our study cohort was diagnosed with breast cancer between 1991 and 2000, and did not receive current standard of care: women with Her2+ tumors did not receive adjuvant trastuzumab, few postmenopausal women received adjuvant aromatase inhibitors. Women entered the WHEL Study on average of 2 years after cancer diagnosis. While we used left-truncated survival models to account for this delayed entry, there could nevertheless have been a selection bias, whereby women who recurred early would not have been eligible to enter our study. Women with the basal, Her2-overexpressed and claudin-low subtypes could have been most susceptible to this selection bias, a possible explanation for the attenuated hazard ratios observed for these groups in our study.

In summary, we confirmed the prognostic value of PAM50 subtypes for breast cancer outcomes in pre- and post-menopausal women in a large independent cohort with 15-year follow-up. Addition of hypoxia signatures further improved prognostication. Relapse and breast cancer mortality rates for women with Luminal B tumors were the highest, especially over the long-term. Future research and clinical trial innovation should focus on this high-risk group.

## Electronic supplementary material

Below is the link to the electronic supplementary material.

Supplementary material 1 (EPS 792 kb)

Supplementary material 2 (DOCX 24 kb)

Supplementary material 3 (DOCX 14 kb)

## Data Availability

The datasets analyzed during the current study are available from the corresponding author on reasonable request.
