# Supplementary material for: Research-based PAM50 signature and long-term breast cancer survival
Source: Breast Cancer Res Treat. 2019 Sep 21;179(1):197–206. doi: 10.1007/s10549-019-05446-y (PMC6985186; doi:10.1007/s10549-019-05446-y)
Supplement: Supplementary file 2 — Supplementary material 2 (DOCX 24 kb) [file 10549_2019_5446_MOESM2_ESM.docx]

Supplemental Table 1: miRGE CodeSet Probes Gene List

| Gene | PAM50 Profile | Hypoxia 1 (VEGF13) | Hypoxia 2 (VEGF15) | Claudin-low | Other | Name |
| --- | --- | --- | --- | --- | --- | --- |
| Signal Transduction | | | | | | |
| AXL |  |  |  | x |  | AXL Receptor Tyrosine Kinase |
| CAV1 |  |  |  | x |  | Caveolin-1 |
| CD24 |  |  |  | x |  | Small cell lung carcinoma cluster 4 antigen |
| DDR1 |  |  |  | x |  | Discoidin domain receptor family, member 1 |
| EGFR | Luminal B |  |  |  |  | Epidermal growth factor receptor |
| EMP3 |  |  |  | x |  | Epithelial Membrane Protein 3 |
| ENO1 |  |  | x |  |  | Enolase 1 |
| ERBB2/HER2 | HER2-enriched |  |  |  |  | Human epidermal growth factor receptor 2 |
| ESR1 | Basal-Like |  |  |  |  | Estrogen Receptor 1 |
| ESRP1 |  |  |  | x |  | Epithelial splicing regulatory protein 1 |
| EVI2A |  |  |  | x |  | Ecotropic viral integration site 2A |
| FGFR4 | HER2-enriched |  |  |  |  | Fibroblast growth factor receptor 4 |
| GNG11 |  |  |  | x |  | G Protein Subunit Gamma 11 |
| GPR160 | Basal-Like |  |  |  |  | G protein-coupled receptor 160 |
| GRB7 | HER2-enriched |  |  |  |  | Growth factor receptor-bound protein 7 |
| LHFP |  |  |  | x |  | Lipoma HMGIC fusion partner |
| MIF |  |  | x |  |  | Macrophage migration inhibitory factor |
| NDRG1 |  | x | x |  |  | N-myc downstream regulated 1 |
| NT5E (CD73) |  |  |  | x |  | 5'-Nucleotidase Ecto |
| PGR | Luminal B |  |  |  |  | Progesterone Receptor |
| RAB25 |  |  |  | x |  | Ras-related protein |
| ROR1 |  |  |  |  | x | Receptor tyrosine kinase like orphan receptor 1 |
| SFRP1 | HER2-enriched |  |  |  |  | Secreted frizzled-related protein 1 |
| SH2B3 |  |  |  | x |  | SH2B adaptor |
| TMEM45B | Basal-Like |  |  |  |  | Transmembrane protein 158 |
| Metabolism | | | | | | |
| ACOT7 |  |  | x |  |  | Acyl-CoA thioesterase 7 |
| ADM |  | x | x |  |  | Adrenomedullin |
| ALDOA |  |  | x |  |  | Aldolase-A, fructose-bisphosphate |
| BLVRA | HER2-enriched |  |  |  |  | Biliverdin reductase A |
| CD68 |  |  |  |  | x | CD68 |
| FABP5 |  | x |  |  |  | Fatty acid binding protein 5 |
| GAL |  | x |  |  |  | Galanin prepropeptide |
| GSTM1 |  |  |  |  | x | Glutathione S-transferase |
| LDHA |  |  | x |  |  | Lactate dehydrogenase A |
| NAT1 | Luminal A |  |  |  |  | N-acetyltransferase 1 |
| NP (PNP) |  | x |  |  |  | Purine nucleoside phosphorylase (PNP) |
| P4HA1 |  |  | x |  |  | Prolyl 4-hydroxylase, alpha-polypeptide I |
| PGAM1 |  |  | x |  |  | Phosphoglycerate mutase I |
| PHGDH | Luminal B |  |  |  |  | Phosphoglycerate dehydrogenase |
| RRAGD |  | x |  |  |  | Ras-related GTP binding D |
| TPI1 |  |  | x |  |  | Triosephosphate isomerase 1 |
| UBE2C | Luminal A |  |  |  |  | Ubiquitin-conjugating enzyme E2C |
| UCHL1 |  | x |  |  |  | Ubiquitin carboxyl-terminal esterase L1 |
| Cell Cycle/Proliferation/Apoptosis | | | | | | |
| BAG1 | HER2-enriched |  |  |  |  | BCL2 associated athanogene |
| BCL2 | Luminal B |  |  |  |  | Bcl-2 |
| BIRC5/Survivin | Luminal A |  |  |  |  | Apoptosis inhibitor survivin |
| CCNB1 | Normal-like |  |  |  |  | Cyclin B1 |
| CCNE1 | HER2-enriched |  |  |  |  | Cyclin E |
| CDC20 | HER2-enriched |  |  |  |  | Cell division cycle 20 homolog |
| CDC6 | Normal-like |  |  |  |  | Cell division cycle 6 homolog |
| CDCA1 (NUF2) | Luminal A |  |  |  |  | NUF2, NDC80 kinetochore complex component, homolog |
| CDKN3 |  |  | x |  |  | Cyclin dependent kinase inhibitor 3 |
| CENPF | Luminal A |  |  |  |  | Centromere protein F |
| CXXC5 | Luminal B |  |  |  |  | CXXC finger protein 5 |
| DDIT4 |  | x |  |  |  | DNA-damage-inducible transcript 4 |
| EXO1 | Luminal A |  |  |  |  | Exonuclease 1 |
| FOXA1 | Basal-Like |  |  |  |  | Forkhead box A1 |
| FOXC1 | Basal-Like |  |  |  |  | Forkhead box C1 |
| GRHL2 |  |  |  | x |  | Grainyhead like transcription factor 2 |
| KI67 (MKI67) | Luminal B |  |  |  |  | Ki 67 |
| KIF2C | Luminal B |  |  |  |  | Kinesin family member 2C |
| KNTC2 (NDC80) | Basal-Like |  |  |  |  | Kinetochore associated 2 |
| MDM2 | Luminal B |  |  |  |  | MDM2, p53 E3 ubiquitin protein ligase homolog |
| MELK | Basal-Like |  |  |  |  | Maternal embryonic leucine zipper kinase |
| MYBL2 | Luminal A |  |  |  |  | v-myb Myeloblastosis viral oncogene homolog (avian)-like 2 |
| MYC | HER2-enriched |  |  |  |  | MYC |
| ORC6L | Luminal B |  |  |  |  | Origin recognition complex, subunit 6 |
| PTTG1 | Luminal A |  |  |  |  | Pituitary tumor-transforming 1 |
| RRM2 | Normal-like |  |  |  |  | Ribonucleotide reductase M21 |
| SCUBE2 |  |  |  |  | x | Signal peptide, CUB domain, EGF-like 2 |
| STK15 (AURKA) |  |  |  |  | x | Aurora kinase A |
| TMEM158 |  |  |  | x |  | Transmembrane protein 158 |
| TYMS | Normal-like |  |  |  |  | Thymidylate synthetase |
| UBE2T | Normal-like |  |  |  |  | Ubiquitin-conjugating enzyme E2T |
| ZEB1 |  |  |  | x |  | Zinc finger E-box binding homeobox 1 |
| Extracellular Membrane Proteins | | | | | | |
| CDH3 | Luminal B |  |  |  |  | Cadherin 3, type 1, P-cadherin (placental)1 |
| CLDN4 |  |  |  | x |  | Claudin 4 |
| DSP |  |  |  | x |  | Desmoplakin |
| EPCAM |  |  |  | x |  | Epithelial cell adhesion molecule |
| F11R |  |  |  | x |  | Junctional adhesion molecule A |
| MIA | Basal-Like |  |  |  |  | Melanoma inhibitory activity |
| MMP11 | Normal-like |  |  |  |  | Matrix metallopeptidase 11 |
| PVRL3 |  |  |  | x |  | Poliovirus receptor-related 3 |
| SLC16A3 |  | x |  |  |  | Solute carrier family 16, member 3 (monocarboxylic acid transporter 4) |
| SLC2A1 |  |  | x |  |  | Solute carrier family 2 member 1 |
| SLC39A6 | Luminal A |  |  |  |  | Solute carrier family 39 (zinc transporter), member 6 |
| TUBB6 |  |  | x |  |  | Tubulin beta-6 |
| Structural | | | | | | |
| ACTR3B | HER2-enriched |  |  |  |  | ARP3 actin-related protein 3 homolog B |
| ANLN | Basal-Like |  |  |  |  | Anillin, actin binding protein |
| CEP55 | Basal-Like |  |  |  |  | Centrosomal protein 55kDa |
| CTSL2 |  |  |  |  | x | Cathepsin L2 |
| FBN1 |  |  |  | x |  | Fibrillin-1 |
| JUP |  |  |  | x |  | Junction plakoglobin |
| KRT14 | Normal-like |  |  |  |  | Keratin 14 |
| KRT17 | Normal-like |  |  |  |  | Keratin 17 |
| KRT19 |  |  |  | x |  | Keratin 19 |
| KRT5 | Normal-like |  |  |  |  | Keratin 5 |
| KRT8 |  |  |  | x |  | Keratin 8 |
| LEPRE1 |  |  |  | x |  | Leprecan-1, Prolyl 3-Hydroxylase 1 |
| MAPT | Luminal A |  |  |  |  | Microtubule-associated protein tau |
| MLPH | Normal-like |  |  |  |  | Melanophilin |
| MPP1 |  |  |  | x |  | Membrane palmitoylated protein 1 |
| MRPS 17 |  |  | x |  |  | Mitochondrial ribosomal protein S17 |
| PLOD1 |  | x |  |  |  | Procollagen-lysine, 2-oxoglutarate 5-dioxygenase |
| SPINT1 |  |  |  | x |  | Serine peptidase inhibitor, Kunitz Type 1 |
| SPINT2 |  |  |  | x |  | Serine peptidase inhibitor, Kunitz Type 2 |
| VAMP8 |  |  |  | x |  | Vesicle associated membrane protein 8 |
| VIM |  |  |  | x |  | Vimentin |
| Angiogenesis | | | | | | |
| ANGPTL4 |  | x |  |  |  | Angiopoietin-like 4 |
| C14ORF58 (FLVCR2) |  | x |  |  |  | Feline leukemia virus subgroup C cellular receptor 2 |
| VEGFA |  | x | x |  |  | Vascular endothelial growth factor A1 |
| Housekeeping | | | | | | |
| B2M |  |  |  |  |  | Beta 2 microglobulin |
| GAPDH |  |  |  |  |  | GAPDH |
| GUS |  |  |  |  |  | Glucuronidase |
| MRPL19 | Housekeeping |  |  |  |  | Mitochondrial ribosomal protein L19 |
| PSMC4 | Housekeeping |  |  |  |  | ATPase 4 |
| PUM1 | Housekeeping |  |  |  |  | Pumilio homolog 1 |
| RPL19 |  |  |  |  |  | Ribosomal protein L19 |
| RPLPO |  |  |  |  |  | Large ribosomal protein |
| SF3A1 | Housekeeping |  |  |  |  | Splicing factor 3a Subunit 1 |
| TFRC |  |  |  |  |  | Transferrin receptor |
| β-actin (ACTB) | Housekeeping |  |  |  |  | β-actin |
