# Supplementary material for: Research-based PAM50 signature and long-term breast cancer survival
Source: Breast Cancer Res Treat. 2019 Sep 21;179(1):197–206. doi: 10.1007/s10549-019-05446-y (PMC6985186; doi:10.1007/s10549-019-05446-y)
Supplement: Supplementary file 3 — Supplementary material 3 (DOCX 14 kb) [file 10549_2019_5446_MOESM3_ESM.docx]

**Table S2: Clinicopathological factors and subtype**

|  | Basal  (% Clinico-pathological) | Her2  (% Clinico-pathological) | LumA  (% Clinico-pathological) | LumB  (% Clinico-pathological) | Normal  (% Clinico-pathological) | Row Total  (% Total N) |
| --- | --- | --- | --- | --- | --- | --- |
| ER-/Her2-/PR+ | 12  (29.3%) | 2  (4.9%) | 21  (51.2%) | 4  (9.8%) | 2  (4.9%) | 41  (3%) |
| ER-/Her2+ | 16  (18.8%) | 52  (61.2%) | 6  (7.1%) | 8  (9.4%) | 3  (3.5%) | 85  (7%) |
| ER or Her2 NA | 5  (22.7%) | 1  (4.5%) | 13  (59.1%) | 2  (9.1%) | 1  (4.5%) | 22  (2%) |
| ER+/Her2- | 34  (4.4%) | 28  (3.6%) | 464  (59.9%) | 224  (28.9%) | 25  (3.2%) | 775  (62%) |
| ER+/Her2+ | 5  (3.8%) | 44  (33.6%) | 38  (29.0%) | 41  (31.3%) | 3  (2.3%) | 131  (10%) |
| Triple Negative | 153  (76.9%) | 12  (6.0%) | 22  (11.1%) | 5  (2.5%) | 7  (3.5%) | 199  (16%) |
| Column Total  (% Total N) | 225  (18.0%) | 139  (11.1%) | 564  (45.0%) | 284  (22.7%) | 41  (3.3%) | 1253  (100%) |
